# Prickly Pear Cacti (*Opuntia* spp.) Cladodes as a Functional Ingredient for Hyperglycemia Management: A Brief Narrative Review

**DOI:** 10.3390/medicina58020300

**Published:** 2022-02-16

**Authors:** Rao Raahim Kashif, Nathan M. D’Cunha, Duane D. Mellor, Natalie I. Alexopoulos, Domenico Sergi, Nenad Naumovski

**Affiliations:** 1 Discipline of Nutrition and Dietetics, Faculty of Health, University of Canberra, Canberra, ACT 2601, Australia; u3149808@uni.canberra.edu.au (R.R.K.);; 2 Functional Foods and Nutrition Research (FFNR) Laboratory, University of Canberra, Bruce, ACT 2617, Australia; 3 Aston Medical School, Aston University, Birmingham B4 7ET, UK; d.mellor@aston.ac.uk; 4 Chiron Organic Health, Wedderburn, VIC 3158, Australia; info@chironorganic.com.au; 5 Department of Translational Medicine, University of Ferrara, 44121 Ferrara, Italy; domenico.sergi@unife.it; 6 Department of Nutrition and Dietetics, School of Health Science and Education, Harokopio University, 17671 Athens, Greece

**Keywords:** prickly pear, cladode, *Opuntia* spp., hypoglycemia, hyperglycemia, type 2 diabetes mellitus, blood glucose

## Abstract

The worldwide prevalence of obesity is increasing along with its comorbidities, including type 2 diabetes mellitus (T2DM). From a pathophysiological perspective, T2DM arises as a consequence of insulin resistance and pancreatic β-cell dysfunction, which together induce chronic hyperglycemia. The pharmacological treatment of T2DM specifically focuses on its management, rather than remission, with a lack of pharmacological agents to prevent the onset of the disease. Considering the role of unhealthy dietary patterns on the development of T2DM, identifying novel food ingredients and bioactive substances may provide new avenues by which to address the T2DM epidemic. In this brief review, we have summarized the latest findings on the consumption of the prickly pear (PP; *Opuntia* spp.) cladode as a potential nutritional tool for the management of hyperglycemia. The consumption of prickly pear cladodes was reported to exert hypoglycemic effects, making it a potential cost-effective nutritional intervention for the management of T2DM. Several studies have demonstrated that the consumption of prickly pear cladodes and the related products reduced post-prandial glucose levels. The cladodes’ high fiber content may be implicated in improving glycemic control, by affecting glucose absorption and effectively slowing its release into the blood circulation. Given these potential hypoglycemic effects, prickly pear cladodes may represent a potential functional food ingredient to improve glycemic control and counter the negative metabolic effects of the modern Western diet. Nonetheless, in consideration of the lack of evidence on the chronic effects of the prickly pear cladode, future research aimed at evaluating its long-term effects on glycemic control is warranted.

## 1. Introduction

The prevalence of obesity is increasing worldwide and continues to represent a major health concern [1], affecting over a third of the global population [2]. Obesity is identified as a major risk factor for a multitude of non-communicable diseases, in particular for the development of cardiovascular disease (CVD) and type 2 diabetes mellitus (T2DM) [3]. Insulin resistance is the link between obesity and T2DM and is characterized as a blunted response of insulin tissue targets to insulin that, in concert with impaired pancreatic β-cell dysfunction, contributes to hyperglycemia [4]. The long-term consequences of untreated hyperglycemia and insulin resistance include the onset and development of diabetic neuropathy, nephropathy, retinopathy, and several other cardiovascular complications [4]. Additionally, T2DM is emerging as a risk factor for several neurodegenerative diseases [5,6]. 

Over 400 million people globally are living with T2DM, and it is one of the fastest-growing global chronic diseases, alongside the obesity epidemic [7,8]. In the US alone, it is the seventh-leading cause of mortality, and over USD 218 billion is spent on its management and other related illnesses that arise as a direct consequence of T2DM [7]. Indeed, besides the health implications, T2DM is also posing a substantial economic burden on healthcare systems. with an increasing demand for more effective therapeutics and preventative tools [9]. However, therapeutic approaches are influenced by the treatment price, accessibility, compliance, and side effects, which may prevent successful outcomes [9,10,11]. Thus, it is imperative to identify strategies by which to prevent the development of this metabolic disease.

**Figure 1:**
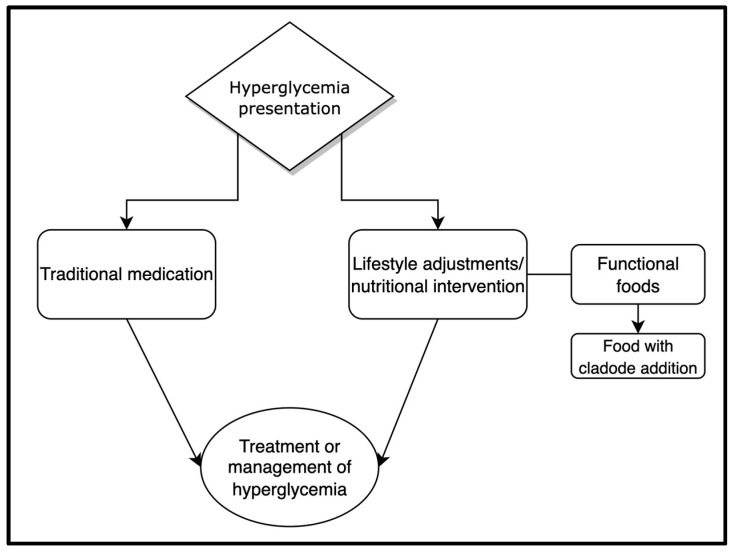
The general approaches of the management and treatment of hyperglycemia, which include the use of traditional medication with/or lifestyle adjustments and nutritional intervention (the use of functional foods) [3,17,26].

It is well established that adequate nutritional interventions can exhibit beneficial outcomes on glycemic control. These include adherence to different dietary patterns, such as the Mediterranean diet [12], and the inclusion of different functional food products as a part of a healthy and balanced diet [3]. Furthermore, the concept of functional foods is a relatively new model that may assist in the management of several health issues, such as hyperglycemia (Figure 1) [13,14,15,16]. Among these functional food products, the cacti (and its products) commonly referred to as “prickly pear” (PP; *Opuntia* spp.) cladodes [17], may represent a promising nutritional approach for the management of hyperglycemia [18,19,20,21]. Although these cladodes have been used as a traditional treatment for T2DM in Mexico [22], the use of *Opuntia* spp. as a basis of functional food products for the management of a range of health conditions has been proposed only relatively recently [23,24,25]. 

A previous systematic literature review published by our research group [17] reported the potential hypoglycemic effects of prickly pear fruit and cladode consumption. Herewith, the main aim of this brief narrative review is to evaluate the current literature pertaining to the potential hypoglycemic effects of cladode consumption, and its potential use in the development of functional foods for the management of hyperglycemia. Furthermore, the potential mechanisms underpinning the effects of the cladodes on glycogenic control will also be explored. 

## 2. Search Strategy and Selection Criteria

The literature search was performed between 15 August 2021 and 20 November 2021, using the following online electronic databases: PubMed, Scopus, Wiley Online Library, and Google Scholar. The searches were conducted using keywords including “diabetes mellitus”, “type 2 diabetes mellitus”, “hyperglycemia”, “hypoglycemia”, “insulin resistance”, “*Opuntia* spp.”, and “prickly pear cladode”. Articles were initially screened by title and abstract, and thereafter by a full-text review to identify which studies to include in the review. For the purposes of this review, we included (1) human case-controlled studies, and (2) articles that focus on the effects of “*Opuntia* spp.”, and “prickly pear cladode” on glucose metabolism and glycemic control. Furthermore, animal trials and in vitro studies were also included, to review the potential mechanisms of action. The literature included in this review was published from 1973 through to 2021.

## 3. Etiology of Type 2 Diabetes

The etiology of T2DM and the associated hyperglycemia can be broadly attributed to the progressive impairment of insulin sensitivity, in concert with the dysfunction of pancreatic β-cells [27,28]. Insulin resistance is largely induced by metabolic inflammation and ectopic lipid accumulation, secondary to defective lipid metabolism and mitochondrial dysfunction [5,29], all of which are associated with obesity [27,30]. The ectopic lipid accumulation and, particularly, of intermediate lipotoxic metabolites, primarily the ceramides, are proposed to be the key mechanisms and metabolites involved in the development of insulin resistance [5]. These lipotoxic metabolites have been implicated in impairing insulin signaling, primarily by hindering protein kinase B (AKT) phosphorylation [27]. Furthermore, a positive energy balance, which underlies obesity, induces an excess of energy stored in adipose tissue in the form of triglycerides. In turn, adipocyte hypertrophy results in adipocyte dysfunction, characterized by the increased secretion of proinflammatory cytokines, such as TNF-α and Il-6, which is further fueled by the infiltration of the adipose tissue by immune cells, including macrophages. This contributes to the low-grade chronic inflammatory status that is typical of obesity and represents a crucial link between obesity and insulin resistance [31]. Although insulin resistance is initially compensated for by insulin hypersecretion once β-cell islet dysfunction arises, this compensatory response eventually becomes compromised, leading to the development of T2DM. 

## 4. Prickly Pear Cacti: General Information and Composition

The PP cacti are a resistant desert species native to the American regions, and, due to their high adaptability, *Opuntia* spp. have spread to other regions around the globe, such as Europe [32] and Australia [13,17,33]. The annual production of PP exceeds 400,000 metric tons in Mexico alone [34], where it is traditionally consumed as a vegetable [32]. Furthermore, the cladodes are typically consumed, whether broiled, blended, or as a juice after the removal of the spines [35,36]. 

The main components in cladodes are carbohydrates (38% dry weight (d.w.)), proteins (11% d.w.) and water (83%; 5:1 biomass to water ratio) [37,38]. The nutritional composition may differ among *Opuntia* spp., dependent on the specimen’s age and environmental factors, such as the cultivation season and geographical position [38]. The primary polysaccharide in cladode is mucilage, an ingredient commonly used in the food industry as an additive and an emulsifier [38]. Furthermore, an analysis of cladode extract indicated the presence of several phytochemicals, mainly polyphenols and phenolic acids [36], all of which have been implicated in certain beneficial health outcomes particularly related to the management of CVD [39,40,41,42]. 

## 5. Anti-Hyperglycemic Effect of the Prickly Pear Cladode

The findings from several different randomized controlled trials (Table 1) have indicated a potential (mainly acute) hypoglycemic effect immediately following the consumption of cladodes. A study conducted by Frati et al. [19] aimed to evaluate the potential hypoglycemic effects of different cladode preparations (500 g; broiled, blended and broiled, blended crude, and heated, blended crude) after ingestion, in participants diagnosed with T2DM (*n* = 8). It was observed that all methods of cladode preparation resulted in an acute reduction of blood glucose at 120 and 180 min following the cladode intake (*p <* 0.01). The peak hypoglycemic effect of the cladode intervention observed a reduction ranging from 23.3 ± 4.4 mg/dL to 25.4 ± 14.3 mg/dL. No difference was noted in the hypoglycemic effectiveness between the preparation methods of the cladode (*p >* 0.05). A similar outcome was also observed in an earlier study by Frati-Munari et al. [43]. In this study, T2DM participants (*n* = 32) were either given 500 g of broiled cladode or 400 mL of water (control). The cladode-ingesting group showed a decrease in blood glucose levels compared to basal values (fasting glycemia), with a mean reduction of 17.6 ± 2.2% of basal values at 180 min after cladode intake. The control group remained the same with respect to their basal blood glucose levels, following the intake of the water (*p >* 0.05). Furthermore, the study also conducted a small separate cross-over trial, where six people with T2DM were each given 500 g of broiled cladode, water, and 500 g of broiled squash on three distinct occasions. When the participants consumed the cladode, a reduction in glycemia was observed, with a mean attenuation of 16.2 ± 1.8% of basal values (fasting glycemia) at 180 min following the intake of the prepared cladode. Conversely, the water and the squash yielded no change in blood glucose levels (*p* > 0.05). 

A study by Frati et al. [18] investigated the cladodes’ acute hypoglycemic effects in diabetic and healthy individuals. Participants with T2DM (*n* = 14) and metabolically healthy participants (*n* = 14) consumed 500 g of cladode. In the T2DM participants, the blood glucose levels reduced by 40.8 ± 4.6 mg/dL less than basal values, at 180 min following the cladode intake (*p* < 0.001, compared to control). However, no change in glycemia was noted in healthy individuals (*p >* 0.05). A study by Frati-Munari et al. [20] involved the consumption of 500 g of cladode by healthy participants (*n* = 16) to assess its hypoglycemic effect. The findings indicated no change in blood glucose levels following the intervention (*p >* 0.05). In light of this finding, it can be concluded that the hypoglycemic effects exerted by cladode consumption become evident only in individuals with impaired glycemic control. 

The longer-term consumption of cladodes was reported in a study by Guavara-Cruz et al. [21], where participants with metabolic syndrome (MetS; *n* = 67) were provided with cladodes (as a part of their regular diet) for two weeks. In this study, both the control and intervention diets consisted of chia seeds, soy protein, and oats with the addition of cladode in the intervention group. The findings of the study indicated an attenuation of relative blood glucose levels (from 21.58 ± 6.39 mmol/L to 19.48 ± 6.39 mmol/L) in the intervention group, which appeared to be consistent throughout the study [21]. However, these results should also be treated with caution, as the contribution of the cladode to the overall hypoglycemic effect could not be inferred because it was used in synergy with other products in the diet.

A study by Linarès et al. [44] used “*NeOpuntia*” capsules (prepared from *Opuntia ficus indica* cladodes) to assess their hypoglycemic effects in participants with MetS (*n* = 68). The study was conducted over a period of six weeks, during which time capsules containing cladode powder were included in a recommended “balanced diet” (2000 kcal; 38% lipids; 17% protein; 45% carbohydrates). The results indicated no change in blood glucose in the group consuming the cladode capsules as part of their “balanced diet” (*p >* 0.05) when compared to a control (*Plantago ovata* seeds). This finding can perhaps suggest that the mechanism by which the cladode exerts its activity may be acute and food matrix-specific. Interestingly, the control group was reported to exhibit increased glycemia during the intervention. Therefore, it can be postulated that the cladode capsules could suppress the hyperglycemic effects of a regular diet. Nevertheless, due to a lack of consensus, the efficacy of cladode on glycemic control is yet to be elucidated. 

An investigation by Godard et al. [45] aimed to assess the acute and chronic effects of cladode consumption in participants with T2DM (*n* = 29). The acute phase of the study occurred as a single bolus of 400 mg OpunDia™ (cladode) or placebo following an oral glucose tolerance testing (OGGT), with a glucose load of 75 g. The results showed a reduction in blood glucose levels in the participants consuming the cladode (*p* < 0.05) at 60 min following intake. The chronic phase of the study involved the regular consumption of a 200 mg bolus of OpunDia™ for 16 weeks. The findings indicated no change in blood glucose levels before and after the intervention (*p* > 0.05). These results support the findings reported by Linarès et al. [44], where no chronic effects of cladode intake on blood glucose levels were demonstrated.

The effects of cladode intake in different breakfast compositions on postprandial blood glucose levels and other relevant metabolic markers were investigated in two acute studies of people with T2DM (*n* = 14) by López-Romero et al. [46]. Two breakfast compositions, a high carbohydrate breakfast (HCB; 300 kcal; 89% carbohydrates; 6% protein; 5% lipids) and a high-soy protein breakfast (HSPB; 344 kcal; 42.4% carbohydrates; 40.7% protein; 16.9 lipids) were given to people with T2DM, with 300 g steamed cladode (treatment) and without (control). The findings of the study showed a reduction in the blood glucose area under the curve of the HCB with steamed cladode compared to only the HCB (287 ± 30 mg/dL to 443 ± 49 mg/dL, respectively; *p* < 0.05). With respect to the HSPB, no difference in postprandial glucose peaks between the cladode and the control group was observed (*p* > 0.05).

## 6. Potential Hypoglycemic Mechanism of Action

**Figure 2:**
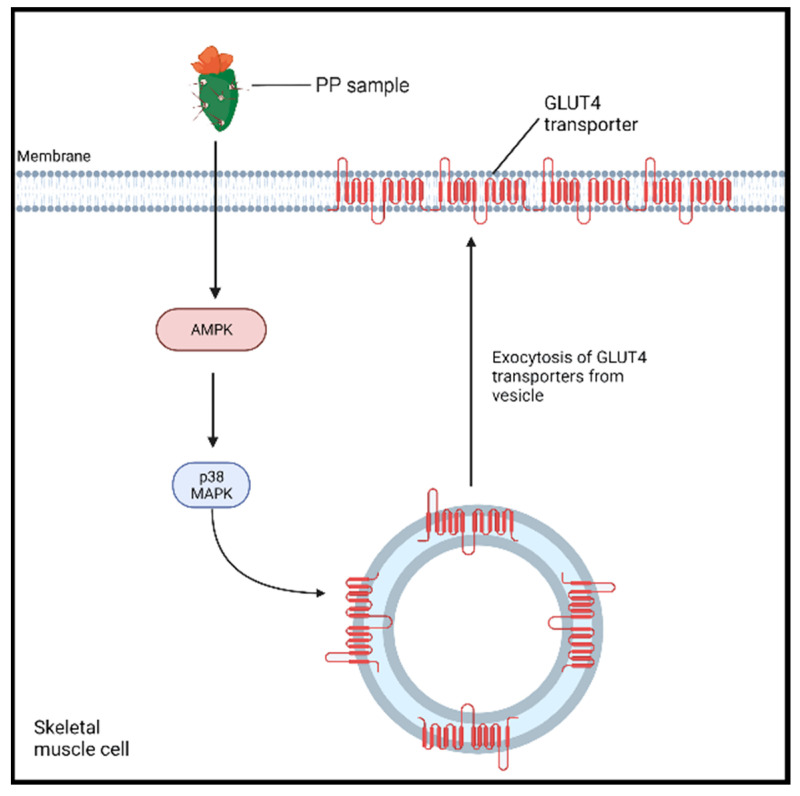
The proposed PP-enhanced AMPK pathway for an antidiabetic effect. PP sample (Opuntia ficus-indica var. saboten (OFS) extract full) enhances the activity of AMP-activated protein kinase (AMPK), which triggers the activation of p38 mitogen-activated kinase (MAPK) [47]. The latter enzyme induces the exocytosis and translocation of GLUT4 to the plasma membrane, enhancing the influx of glucose into the cell.

Cladode consumption has been observed to exert acute hypoglycemic effects [17,18,19]; however, the exact mechanisms of its action are still not completely elucidated [17]. A study conducted by Leem et al. [47] investigated the underlying mechanism of the in vitro antidiabetic effect of *Opuntia ficus indica* var. *saboten* cladode powder extract in rats with L6 myoblasts [47]. The addition of the extract produced an increase in adenosine monophosphate-activated protein kinase (AMPK) and p38 mitogen-activated kinase (MAPK) activities (*p <* 0.05) within the myoblasts (Figure 2) compared to control cells (100 nM insulin). The latter enzymes are implicated as cellular energy sensors that promote the transport of glucose to skeletal muscle. The activation of AMPK promotes its interaction with p38 MAPK, which, in turn, induces the translocation of glucose 4 transporters (GLUT4) to the cell membrane [47,48]. This causes an increase in the available GLUT4 transporters on the plasma membrane, leading to an 11.7% increase in glucose uptake following the treatment when compared to a control. To confirm these findings, AMPK and p38MAPK were inhibited, which largely abolished the effects of the cladode extract treatment on the L6 myotubes’ glucose uptake. Interestingly, AMPK activation noticeably protects against hepatic lipotoxicity, a known contributor to the pathogenesis of T2DM, in cultured hepatocytes and animal liver [49].

A study by Sanchez-Tapia et al. [50] investigated the effects of cladode consumption in obese rats that were fed a high-sucrose diet. It was observed that supplementation with cladodes improved rat metabolic health, despite the animals being fed a high-fructose and sucrose (HDS) diet [50,51]. Indeed, the supplementation of cladode in the diet attenuated triglycerides, total cholesterol, and Gastric Inhibitory Polypeptide (GIP), and promoted an improvement in glycemic control and insulin sensitivity. Additionally, the Opuntia ficus-indica var. saboten (OFS) diet, when supplemented with nopal, led to an increased abundance of the alpha-diversity genus (mainly the *Prevotella* genus) of gut microbiota by up to 11.6-fold compared to the control group [50]. Members of the *Prevotella* genus play a potential role in enhanced glucose metabolism by promoting increased glycogen storage [52]. The addition of cladode to the OFS diet also caused a reduction in serum GIP and insulin. High concentrations of GIP are associated with high lipopolysaccharides (LPS) [3]. An in vitro study also proposed that LPS causes an inflammatory response that subsequently hinders glucose transport in the myotubules [53]. It was suggested that high LPS may potentially contribute to the pathogenesis of hyperglycemia. Therefore, the findings of the study by Sanches-Tapia et al. [50] suggest that the consumption of cladodes may regulate the synthesis of LPS, and thereby prevent hyperglycemia. Nevertheless, it is important to note that the links between in vitro studies and the effects observed in humans are still very speculative. 

The modulation of the gut microbiota and its contribution to metabolic disease is well established, and detailed information is described elsewhere [54,55]. Some of the potential mechanisms underlying the regulation of blood glucose metabolism can also be ascribed to the gut microbiota via the modulation of inflammation, gut permeability, and insulin sensitivity in several mammalian models. For example, while intestinal permeability-induced endotoxemia may contribute to the pathogenesis of T2DM, some bacterial species (*Bacteroides vulgatus* and *B. dorei*) have been reported to reduce gut permeability in animal models [55,56]. This, in turn, contributes to a decrease in circulating endotoxins like LPS, and systemic chronic inflammation, a key pathogenetic feature of insulin resistance [57]. In this regard, the prebiotic effects of cladodes and the consequent modulation of the gut microbiota [58] may also contribute to its effect on glycaemic control [59]. However, the fact must not be overlooked that the majority of evidence on the hypoglycemic effects of cladodes comes from acute studies, and it is, therefore, implausible that they may rely on the modulation of the gut microbiota. Nonetheless, the prebiotic effect of the cladode and its potential effects on glycemic control via the gut microbiota remains a field worthy of further investigation.

Moreover, the reduction of blood glucose by cladode consumption may be due to its high dietary fiber content [17]. The consumption of water-soluble dietary fiber has the ability to retard digestion, which subsequently slows down the absorption of sugars [60], preventing circulating glucose peaks. The presence of dietary fibers was confirmed in an extract analysis of fresh nopal by Hwang et al. [61]. It was observed that the cladode dry powder and water extract contained 4.99 ± 0.42% and 45.92 ± 5.17% of soluble dietary fiber, respectively. 

## 7. Prickly Pear Cladode as a Functional Ingredient for Hyperglycemia Management

Foods are no longer only being consumed to promote satiety or meet nutritional requirements but are increasingly being used to potentially prevent or manage chronic diseases in the form of functional foods [62,63]. A food is deemed to be functional if it constructively improves the health of an individual beyond its “normal” nutritional value [64]. These food products are also becoming more popular, as is evident from a global market share now estimated to be over USD 180 billion, with an 8% annual growth [65]. As such, new and/or improved functional foods are becoming a valuable commodity of economic and health interest alike. 

With the general increase in the popularity of functional foods, the application of cladodes in different food products could be seen as advantageous due to their potential beneficial health properties. One of the proposed food candidates for cladode addition is pasta, a popular dish consumed by many around the world. A survey of an Italian population sample, performed by Palmieri et al. [66], highlighted considerable interest in the health benefits and nutritional value of functional pasta containing *Opuntia* powder, as long as the pasta retained a “familiar” pasta taste [66]. Hence, any cladode powder used in the production of functional pasta should attempt to retain similar organoleptic properties and appearance to traditional durum wheat-based pasta. This will avoid the familiarity concern issue in the survey. Furthermore, a pilot study conducted by Aiello et al. [67] demonstrated the effectiveness of functional cladode-fortified pasta to manage hyperglycemia. The study involved 42 healthy individuals who consumed 500 g/week of functional pasta (with 3% of cladode extract) for four weeks [64]. The findings indicated an attenuation of fasting serum glucose levels from 84.02 ± 10.59 mg/dL to 80.89 ± 10.62 mg/dL (*p <* 0.05), which was attributed to the functional pasta. Therefore, cladodes represent a potential addition to functional pasta, to prevent or treat hyperglycemia. 

Some of the other food products where cladode powder can be utilized include using cladode flour as a substitute for plain flour in gluten-free crackers. A study by Dick et al. [68] reported a higher total phenolic content in gluten-free cladode-fortified crackers (increased from 0.26 ± 0.02 to 0.80 ± 0.02 mg_GAE_/g d.w.) (*p <* 0.05) in comparison to the unfortified gluten-free crackers [68]. Interestingly, preliminary evidence in clinical trials has demonstrated an association between dietary polyphenols and blood glucose control; however, these mechanisms are still unexplored [69]. 

A study conducted by Msaddak et al. [70] investigated the effects of cladode powder addition, at varying concentrations, on bread production. The addition of cladode powder at 5% was sufficient to significantly increase (*p <* 0.05) the total phenolic content from 0.90  ±  0.01 to 5.22  ±  0.02 mg_GAE_ per 100 g of bread. Furthermore, bread containing cladode powder also showed higher antioxidant activity, while there were no major changes to the traditional organoleptic properties [70]. Other proposed foods where the addition of PP may be beneficial include cereals, fortified milk, and wine [17,70]. The potential for cladodes as a beneficial ingredient in functional foods remains one of the most significant areas of research and innovation and warrants further investigation.

## 8. Adverse Effects of Prickly Pear Consumption

The reported adverse effects of cladode consumption in the literature remain scarce. Nonetheless, a documented case study of low colonic obstruction in a patient was attributed to PP seed intake. Furthermore, a systematic review investigating gastrointestinal seed bezoar cases revealed that 28 individuals had phytobezoars induced by PP consumption [71]. A bezoar is a persistent indigestible mass accumulating within the gastrointestinal tract, with the most common presenting symptoms being rectal pain, intestinal obstruction, and constipation [72]. Although the occurrence of PP-induced bezoar is rare, individuals should be vigilant if any pertinent symptoms arise, especially given the association of T2DM with gastroparesis. A few anecdotal cases have been reported, with subjects presenting with chronic diarrhea and encopresis that was attributed to PP intake [73]. It would also be advisable that individuals with low blood glucose may want to abstain from PP consumption as it may exacerbate their hypoglycemia.

## 9. Future Perspectives

The current literature regarding the cladode and its hypoglycemic effects suggests that it is a promising ingredient for the management of glycemia. However, current evidence for these potential health benefits remains relatively scarce and mostly relies upon acute studies. Future research should investigate the efficacy of cladodes upon individuals with prediabetes, as the current literature has largely excluded this group [17,18,19]. In this context, it is of pivotal importance to determine whether cladodes can prevent or slow down the progression toward overt T2DM. Furthermore, the mechanism by which the cladode exerts its hypoglycemic effect differs or may vary depending on the glycemic status of the individual. This was inferred by a lack of change in terms of glycemia in healthy individuals, upon consumption of the cladode. Future research should also attempt to use a long-term study design as this part of the literature remains vague. Some studies analyzing the long-term hypoglycemic effects of cladodes have not used the product in isolation, but rather in synergy with other dietary items [21,46]. As such, the level of contribution of the cladode to the effects of the intervention cannot be accurately determined. Another future avenue for research is the analysis of the bioactive, phytochemicals, nutritional profile, and rheology of functional pasta or any other functional food products supplemented with cladode. Lastly, the potential mechanisms underpinning cladodes’ hypoglycemic effects are not well understood, and therefore require further investigation.

## 10. Conclusions

The findings of this brief narrative literature review suggest the use of cladode as a potential adjunct treatment for T2DM. Several studies showed reductions in blood glucose levels in individuals following the intake of cladode, particularly in an acute study design. The mechanism(s) by which the cladode exerts its hypoglycemic activity remains undefined; however, various mechanisms have been proposed. These include a cladode-induced AMPK pathway, ultimately stimulating the translocation of GLUT4 transporters to the membrane. Another mechanism by which cladode exerts its activity is by affecting the gut microbiota, ultimately altering the glucose metabolism to reduce glucose peaks. Additionally, the nopal may potentially suppress the inflammatory effect of LPS, thereby inhibiting LPS-induced insulin resistance and glucose intolerance. Given its potential anti-hyperglycemic effect, the use of PP in the production of functional foods, such as functional pasta, may represent a promising nutritional tool to manage hyperglycemia. 

## Figures and Tables

**Table 1:** Studies and their respective details investigating the hypoglycemic effect of cladode.

Reference	Aim/s	Participants	Intervention/Design	Results	
Frati et al. [19]	To assess the effect of *Opuntia ficus indica* cladode on hyperglycemia in T2DM subjects.	T2DM participants (*n* = 8; 2 M and 6 F; mean age: 55 years)	Length: Acute (single consumption) Study design: Cross-over trial Treatment: 500 g of cladode given to fasted (12 h) subjects. Cladode prepared as broiled, blended, crude, and heated (60 °C) crude. Measurements: GLU at 40, 60, 120, 180 min following intervention.	Reductions in GLU (*p* < 0.01) reached at 120 and 180 min. Major hypoglycemic effects shown after cladode consumption ranged from 23.3 ± 4.4 to 25.4 ± 14.3 mg/dL. No difference in the hypoglycemic effects between cladode preparations (all *p* > 0.05).	
Frati et al. [43]	To investigate the effect of *Opuntia streptacantha* cladode on hyperglycemia in T2DM subjects.	Group 1: T2DM participants (*n* = 16; 9 M and 7 F; mean age: 43.8 ± 11.4 years) Group 2: T2DM participants (*n* = 10; 6 M and 4 F; mean age: 46.2 ± 10.8 years) Group 3: T2DM participants (*n* = 6; 4 M and 2 F; mean age: 48.0 ± 11.7)	Length: Acute (single consumption) Study design: Randomized control-trial Treatment (fasted 12 h): Group 1: 500 g of broiled cladode. Group 2: 400 mL of water. Group 3: 500 g of broiled cladode (test 1), 400 mL of water (test 2), 500 g of broiled squash (zucchini) (test 3) Measurements: GLU at 0, 60, 120, 180 min following intervention.	Group 1: Reduction in GLU (*p* < 0.001) with mean reduction of 17.6 ± 2.2% of basal value at 180 min. Group 2: No change in GLU (*p* > 0.05). Group 3: Test 1—reduction in GLU (*p* < 0.001) with mean reduction of 16.2 ± 1.8% of basal value at 180 min; test 2, 3—no change in GLU (*p* > 0.05).	
Frati et al. [18]	To evaluate the acute hypoglycemic effect of *O. streptacantha Lem.* intake in “healthy” and diabetic individuals.	Group 1: T2DM participants (*n* = 14; 9 M and 5 F; mean age: 43 years; age range: 36–65 years). Group 2: “Healthy” participants (*n* = 14; 9 MJ and 5 F; mean age: 33 years; age range: 15–45 years)	Length: Acute (single consumption) Study design: Randomized control trial Group 1 and 2 treatments: 500 g steamed cladode or 400 mL water (placebo) given to fasted subjects. Measurements: GLU, INS at 0, 60, 120, and 180 min following the intervention.	Group 1: Reduction in GLU (60 min: *p* < 0.005; 120 min: *p* < 0.005; 180 min: *p* < 0.005) reaching 40.8 + 4.6 mg/dL less than basal value. Reduction in INS (120 min: *p* < 0.005; 180 min: *p* < 0.005) reaching 7.8 + 1.5 µU/mL less than basal value. Group 2: No change in GLU and INS (*p* > 0.05).	
Frati et al. [20]	To assess the acute hypoglycemic effect of *O. streptacantha Lem.* intake in “healthy” adults.	“Healthy” participants (*n* = 16) Group 1: (*n* = 5) Group 2: (*n* = 6) Group 3: (*n* = 5)	Length: Acute (single consumption) Group 1: 12 hr fasted + 100 g of cladode Group 2: OGTT (25 g GLU load), 100 g of cladode given after time 0, before GLU load. Group 3: OGTT (25 GLU load) + 100 g of cladode Measurements: GLU, INS at 0, 30, 60, 120 and 180 min following intervention.	Group 1: Attenuation of GLU at 60 min; 180 min (*p* < 0.025). No change in INS (*p* > 0.05). Group 2, 3: No change in GLU, INS (*p* > 0.05).	
Guevara-Cruz et al. [21]	To investigate the effect of dietary patterns, featuring nopal cladode, on biochemical markers (GLU, INS).	MetS participants (*n* = 67; age: 20–60 years; satisfied 3 positive criteria for MetS).	2 weeks prior to treatment: Participants were put on a reduced energy diet, low saturated fat, and low cholesterol diet (50–60% CH, 15% PRO and 25–35% fat). Treatment: Length: 2 months Study design: Single-center, randomized, placebo-controlled, double-blind, parallel-arm study. Group 1: Controlled dietary pattern Group 2: Placebo Dietary pattern: 100 g of cladode, 4 g of chia seeds, 22 g of oats, 32 g of soybean proteins, 0.02 g of sweetener, and 1 g of flavoring. Placebo: 30 g of calcium caseinate, 30 g of maltodextrin, 0.02 g of sweetener and 1 g of flavoring. Pre/post measurements: GLU, INS.	Group 1: Reductions in GLU AUC (from 388.8 ± 115.2 mg/dL to 351.0 ± 115.2 mg/dL), and in AUC INS (from 26.4 ± 14.4 ng/mL to 17.4 ± 10.4 ng/mL) (*p* < 0.0001). Group 2: No difference in GLU, INS (*p* > 0.05).	
Linarès et al. [44]	The study aimed to evaluate “NeOpuntia” on blood lipid parameters and MetS, including glycemia	MetS participants (*n* = 59; 0 M and 59 F; age distribution: 10.29% <35, 27.94% 35 to 45, 41.18% 45 to 55 and 29.59% >55; mean age: 47.3 ± 10.1 years) Group 1: Treatment (*n* = 35) Group 2: Placebo (*n* = 33)	Length: 6-weeks Study design: Monocentric, randomized, double-blind, placebo-controlled study Group 1: balanced diet (45% CH, 17% PRO and 38% fats; 2000 kcal), 3 x “NeOpuntia” capsule after meals/day. Group 2: balanced diet (45% CH, 17% PRO and 38% fats; 2000 kcal), 3 x placebo capsule after meals/day. Measurements: GLU at day 1, day 14 and day 42.	Group 1: Treatment group remained at the same GLU level. Group 2: Increase in GLU.	
Godard et al. [45]	To assess the acute and hypoglycemic effect of OpunDia™ (*O. ficus indica*) in obese and pre-diabetic individuals.	Pre-diabetic and obese participants (*n* = 29; age: 20–50 years) Group 1: Treatment (*n* = 15) Group 2: Placebo (*n* = 14)	Length: Acute phase (single consumption) and chronic phase (16-weeks) Acute phase: Group 1: 400 mg bolus of OpunDia™ 30 min before OGGT (75 g GLU load). Group 2: 400 mg of the placebo 30 min before OGGT (75 g GLU load). Pre/post measurements: GLU Chronic phase: Group 1: 16-week supply of 200 mg OpunDia™ Group 2: 16-week supply of the placebo Pre/post measurements: GLU	Acute phase: Reductions in GLU in the treatment compared to placebo at 60 (205.92 ± 36.90 and 188.84 ± 38.43 mg/dL respectively), 90 (184.55 ± 33.67 and 169.74 ± 35.16 mg/dL respectively) and 120 min (159.24 ± 17.85 and 148.89 ± 24.86 mg/dL respectively) (*p* < 0.05). Chronic phase: No difference in GLU (*p* > 0.05)	
López-Romero et al. [46]	To investiage the effect of nopal in breakfast (2 compositions) upon metabolic markers in T2DM and “healthy” individuals	Study 1: “healthy” participants (*n* = 4; 3 M and 4 F; mean age: 20.6.3 ± 1.2 years; mean BMI: 23.05 ± 0.8). Study 2: T2DM participants (*n* = 14; 4 M and 10 F; mean age: 48.0 ± 2.1; mean BMI: 28.9 ± 1.0; glycosylated hemoglobin levels mean: 6.5 ± 0.2%)	Study 1: Length: Acute (single consumption) Group 1 (treatment): 50 g of dehydrated nopal. Group 2 (placebo): 50 g of available carbohydrates from GLU. Study 2: Length: Acute (single consumption) Group 1 (treatment): High CH breakfast (HCB) or high soy-protein breakfast (HSBP) with or without (random) 300 g steam nopal. Group 2 (placebo): HCB or HSBP. HCB: 300 kcal, 89% CH, 6% PRO, 5% fat in apple juice (240 mL), white bread (55.6 g) and strawberry jam (21 g). HSP: 344 kcal, 42.4% CH, 40.7% PRO, 16.9% fat in soy hamburger (61.5 g) and soymilk beverage (230 mL) Pre/post measurements: GLU, Glycemic index, insulinemic index, glucagon-like peptide 1 (GIP-1) index.	Study 1: Glycemic index is 32.5 ± 4.0, insulinemic, Gastric Inhibitory Polypeptide index 6.5 ± 3.0, and GLP-1 index was 25.9 ± 18.0. Study 2: Group 1: Reduction in GLU AUC of HCB + nopal compared to only HCB (287 ± 30 and 443 ± 49 respectively). Reduction in GLU peaks HSPB + nopal at 30 min and 45 min (*p* < 0.05).	

Key: GLU: blood glucose; INS: insulin; AUC: area under the curve; OGGT: oral glucose tolerance test; BMI: body mass index CH: carbohydrate; PRO: protein; M: males; F: females.

## Data Availability

Not Applicable.
